# Consensus of the Present and Prospects on Endoscopic Diagnosis and Treatment in East Asian Countries

**DOI:** 10.1155/2012/808365

**Published:** 2012-10-10

**Authors:** Takeshi Kamiya, Takashi Joh, Jose D. Sollano, Qi Zhu, Udom Kachintorn, Abdul Aziz Rani, Ki-Baik Hahm, Shin'ichi Takahashi, Yoshikazu Kinoshita, Takayuki Matsumoto, Yuji Naito, Koji Takeuchi, Tetsuo Arakawa, Akira Terano

**Affiliations:** ^1^Department of Gastroenterology and Metabolism, Graduate School of Medical Sciences, Nagoya City University, 1 Kawasumi, Mizuho-cho, Mizuho-ku, Nagoya 467-8601, Japan; ^2^Department of Medicine, University of Santo Tomas, Manila, Philippines; ^3^Department of Gastroenterology, Rui Jing Hospital, Shanghai Jino Tong University School of Medicine, Shanghai, China; ^4^Division of Gastroenterology, Department of Internal Medicine, Faculty of Medicine, Siriraj Hospital Mahidol University, Bangkok, Thailand; ^5^Department of Internal Medicine, Faculty of Medicine and Cipto Mangunkusumo Hospital, University of Indonesia and Indonesian University School of Medicine, Jakarta, Indonesia; ^6^Lee Gil Ya Cancer and Diabetes Institute, Gachon University of Medicine and Science, Incheon, Republic of Korea; ^7^Department of Gastroenterology, School of Medicine, Kyorin University, Tokyo, Japan; ^8^Second Department of Internal Medicine, Shimane University School of Medicine, Izumo, Japan; ^9^Division of Lower Gastroenterology, Department of Medicine, Hyogo College of Medicine, Nishinomiya, Japan; ^10^Department of Molecular Gastroenterology and Hepatology, Kyoto Prefectural University of Medicine, Kyoto, Japan; ^11^Division of Pathological Sciences, Department of Pharmacology and Experimental Therapeutics, Kyoto Pharmaceutical University, Kyoto, Japan; ^12^Department of Gastroenterology, Graduate School of Medicine, Osaka City University, Osaka, japan; ^13^Dokkyo Medical University, Mibu, Shimotsuga, Japan

## Abstract

*Background and Aim*. New diagnostic or therapeutic methods in endoscopy have been used. Current clinical application of these procedures is not well known. The aim of this study is to investigate the present situation on endoscopic diagnosis and treatment of gastrointestinal disorders in East Asian countries. *Method*. A representative member from the International Gastrointestinal Consensus Symposium Committee provided a questionnaire to physicians in China, Indonesia, Japan, Korea, the Philippines, and Thailand. *Results*. In total, 514 physicians including gastroenterologists, surgeons, and general practitioners enrolled. The most frequently occurring disorder as the origin of upper gastrointestinal bleeding is gastric ulcer. Capsule endoscopy is selected as the first choice for the diagnosis of small intestine bleeding. The second choice was double-balloon endoscopy or angiography. For patients with gastric adenoma, the number of physicians who choose endoscopic mucosal resection is larger than those selecting endoscopic submucosal dissection (ESD) in China, Indonesia, the Philippines, and Thailand. ESD is chosen first in Japan and Korea. *Conclusion*. New instruments or techniques on endoscopy have not come into wide use yet, and there is diversity in the situation on it in Asian countries. We should unify the endoscopic diagnostic criteria or treated strategy in patients with GI disease.

## 1. Introduction

Recently, new diagnostic methods in endoscopy and novel endoscopic therapeutic techniques have been used. For example, capsule endoscopy (CE) [1, 2] and double-balloon endoscopy (DBE) [3–6] are new technologies that have been developed for the investigation of the small bowel. Endoscopic submucosal dissection (ESD) [7–10] has been used as a method for the endoscopic ablation of early cancer of the esophagus, stomach, and colon. However, the current clinical application of these new endoscopic procedures and techniques in East Asian countries is not well known.

 In this survey, the authors investigated the present situation on endoscopic diagnosis and treatment of gastrointestinal disorders in East Asian countries by questionnaire, ascertaining the latest information on digestive endoscopy. In addition, the differences in diagnostic tools and patient management that exist between countries from a physician perspective were analyzed. 

## 2. Subjects and Methods

### 2.1. Subjects

Gastroenterologists, surgeons, and general practitioners in China, Indonesia, Japan, Korea, the Philippines, and Thailand participated in this survey. All subjects work in an urban environment. They were asked to complete a questionnaire concerning the present situation and future prospects on endoscopic diagnosis and treatment in Asia.

### 2.2. Methods

This is the third East Asian questionnaire-based survey done by the International Gastrointestinal Consensus Symposium (IGICS), which is the international section of the Japanese Gastroenterological Association. A representative person from each country was selected from the Committee of IGICS, and IGICS Committee sent the questionnaire to them by e-mail. A representative member provided this questionnaire to physicians working in a university, hospital, or clinic in each country by mail or e-mail, starting at the beginning of July 2009. Responses were collected until the end of December 2009. The questionnaire focused on the following items: (1) endoscopic experience and performance by each physician; (2) present situation on endoscopic diagnosis of several diseases in the esophagus, stomach, small intestine, and colon; (3) first-line endoscopic treatment for some common gastrointestinal (GI) diseases; (4) the clinical application of new instruments and novel therapeutic techniques. The contents of the questionnaire are described in the Appendix. 

## 3. Results

### 3.1. Participants

In total, 514 physicians enrolled in this study. The number of physicians participating in this survey was the largest in Japan and the lowest in the Philippines. However, there were no significant differences among all countries. Three-fourths of participants practice gastrointestinal internal medicine, and the others were surgeons. The male/female ratio was 444/70.

### 3.2. The Endoscopic Experience

**Figure 1:**
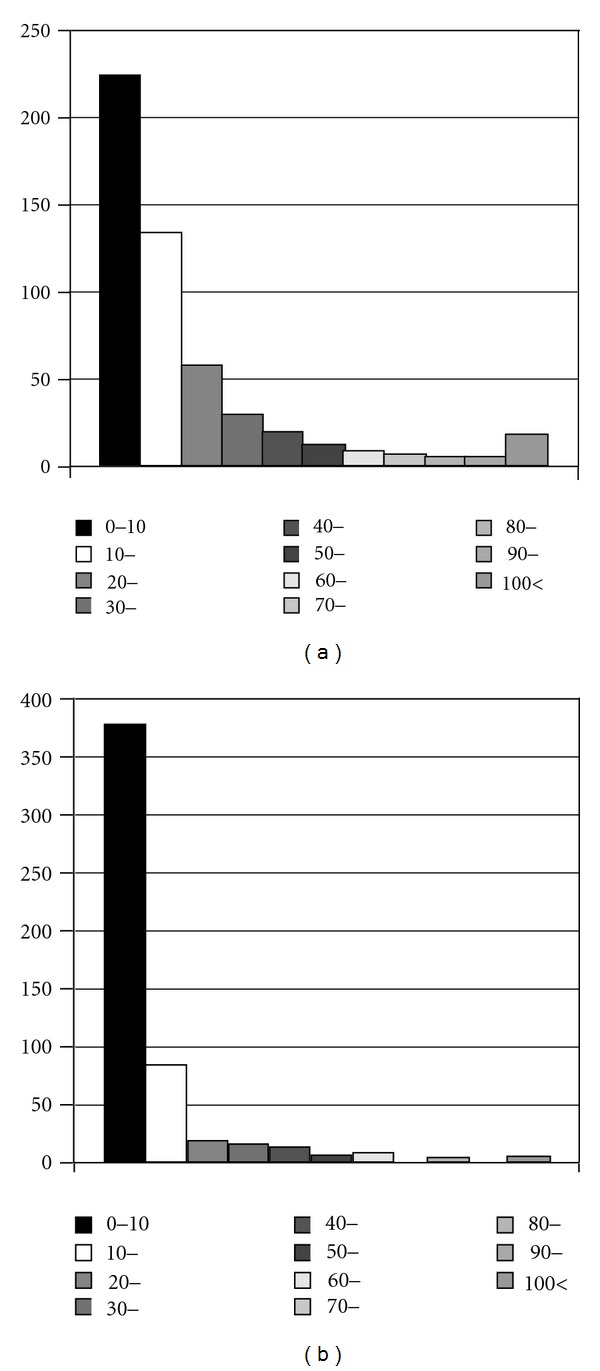
(a) The number of performances of upper gastrointestinal endoscopy in a week. (b) The number of performance of colonoscopy in a week.

**Figure 2:**
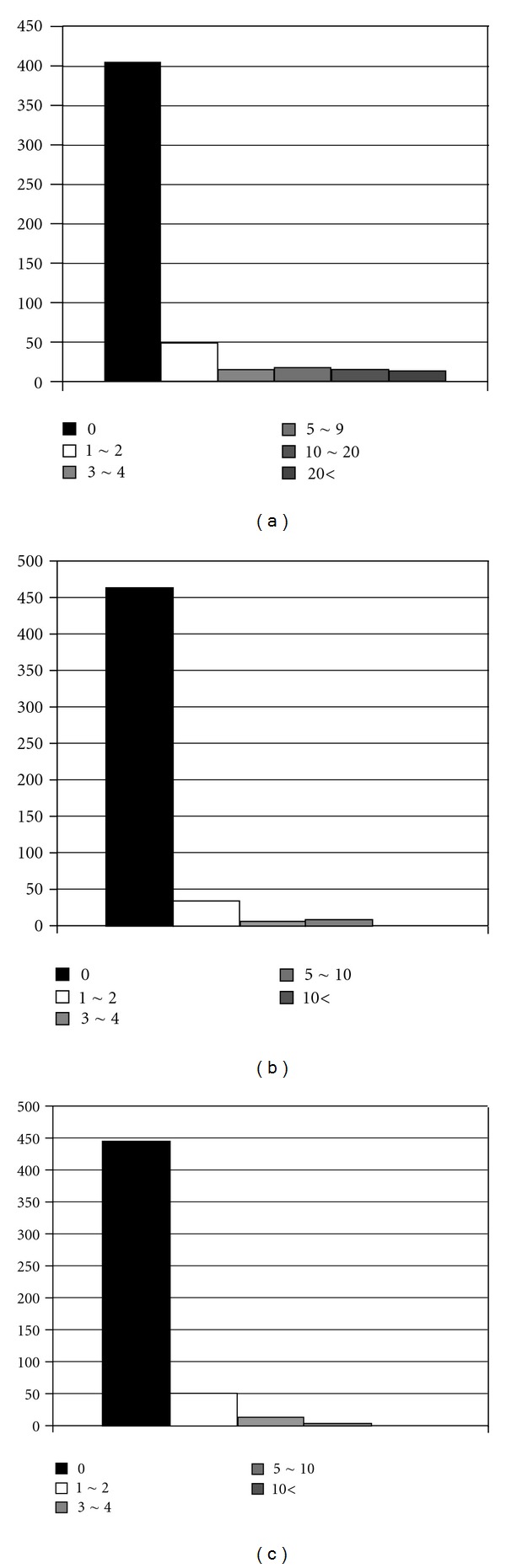
(a) The number of performances of ultrasonography (EUS) in a month. (b) The number of performances of double-balloon endoscopy in a month. (c) The number of performances of capsule endoscopy in a month.

Table 1 shows the duration of endoscopic experience by participating physicians in each country (Table 1). The peak was less than 5 years in 4 countries except Japan and Thailand. The duration in more than half of physicians was less than 10 years. Three hundred seventy-seven physicians answered 0–10 in performance of upper GI endoscopy in a week. Almost 80% of them performed it less than 30 times weekly (Figure 1(a)). In the case of colonoscopy, 90% of respondents perform it under 30 times a week (Figure 1(b)). The number of performances of endoscopic ultrasound (EUS) was smaller compared to upper GI endoscopy or colonoscopy (Figure 2(a)). Ninety percent of subjects answered zero in performance of both CE and DBE (Figures 2(b), and 2(c)).

### 3.3. The Diagnosis of Upper Gastrointestinal Disorders

The first disorder diagnosed by upper GI endoscopy was gastritis in all 6 countries. The second was reflux esophagitis or gastric ulcer (Table 2(a)). The most frequently occurring disorder as the origin of upper GI bleeding was gastric ulcer. However, duodenal ulcer was the first in China and esophageal varices in the Philippines (Table 2(b)).

### 3.4. The Diagnosis of Lower Gastrointestinal Disorders

The frequent disorders diagnosed by colonoscopy were colon polyps and colorectal cancer (Table 2(c)). The first disorder as the origin of lower GI bleeding was hemorrhoid in all countries. The second disease was colorectal cancer in 5 countries with the exception of Japan. In Japan, diverticulum was observed as the second most occurring. Overall, colorectal cancer, ulcerative colitis, ischemic colitis, diverticulum, and polyps were almost equal as the third most frequent disorder of origin of lower GI bleeding (Table 2(d)). 

CE was selected as the first choice for the diagnosis of small intestine bleeding. The second choice was DBE or angiography. However, in this questionnaire, 34 items were left blank (Table 2(e)). 

**Figure 3:**
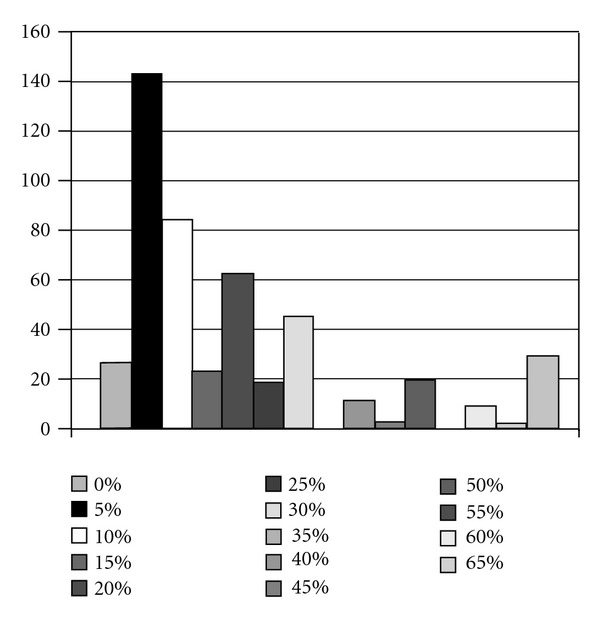
The percentage of patients who could not find the origin of gastrointestinal bleeding endoscopically.

There were a variety of answers in question 12 from 0 to more than 75%. The peak can be seen at 5 and 10% (Figure 3). 

### 3.5. Endoscopic Treatment on Gastrointestinal Disorders

Endoscopic variceal ligation (EVL) was used as first choice to treat the patients with esophageal varices by physicians in all five countries, but S-B tube was selected first in China. Endoscopic injection sclerotherapy (EIS) was selected as the second in Japan, Indonesia, the Philippines, and Thailand (Table 3(a)). For patients with gastric adenoma 2 cm in diameter, the number of physicians who choose endoscopic mucosal resection (EMR) is larger than those selecting ESD in China, Japan, the Philippines, and Thailand. ESD was chosen first by physicians in Japan and Korea. However, the number of those who choose ESD and EMR was nearly equal among Japanese physicians (Table 3(b)). 

The most often used endoscopic therapy of patients with bleeding from exposed vessel in gastric ulcer was clipping in 4 countries. Local injection of hypertonic saline plus epinephrine (HSE) therapy was selected first in Indonesia and the Philippines (Table 3(c)). Another treatments such as electrocoagulation, heat probe, and intravenous proton pump inhibitor (PPI) injection were almost equally selected.

In the case of colonic flat adenoma 3 cm in diameter, a difference in preferred treatment was observed among the countries. ESD was chosen first in Japan and Korea, and EMR was selected in the Philippines and Thailand. On the other hand, surgical operation was the first choice in China and Indonesia (Table 3(d)). There were 37 and 29 blanks in question 14 and 18, respectively. Furthermore, almost 70% of respondents answered zero to both questions 16 and 20, indicating they do not perform endoscopic treatment to early cancer of the stomach and colon. 

### 3.6. The Definition of Early Gastric or Colonic Cancer

Almost all physicians agreed the concept of early gastric or colonic cancer, defined as tumor cells localized within the mucosal or submucosal layer, and not invading the muscular layer or under (data not shown).

## 4. Discussion

This is the first multicountry, East Asia study to examine the present situation on gastrointestinal endoscopic diagnosis and treatment. The authors provide a point-by-point discussion of the diagnosis and first-line endoscopic therapy of several gastrointestinal diseases, and clinical application of novel therapeutic techniques on endoscopy based on the responses of physicians to a questionnaire-based survey. 

As for endoscopic experience, over 80% of physicians perform both upper gastrointestinal endoscopy and colonoscopy less than 30 times a week. These results reflect that there may be many residents or trainers in this survey. 

The frequency of EUS performance is much lower compared to upper GI endoscopy or colonoscopy. EUS is one of the fastest growing areas within GI endoscopy as well as pancreatobiliary lesion. It has been utilized for assessing cancer stage and evaluation of submucosal lesions. However, EUS may not be considered a routine examination yet. Ninety percent of physicians answered zero in performance of DBE and CE. These results were not expected. 

The most frequent disorder diagnosed by upper gastrointestinal endoscopy is gastritis in all 6 countries. One reason of this result is that the prevalence of *Helicobacter pylori* (*HP*) infection is still high in East Asian countries. In addition, these patients of gastritis probably include functional dyspepsia patients, who have chronic upper GI symptoms with no mucosal lesion on endoscopy. Gastritis will be on the decline with the improvement of hygiene and decrease in *HP* prevalence in future. The second disorder diagnosed is reflux esophagitis in 3 countries. The prevalence of gastroesophageal reflux disease (GERD) in Asian countries is considered to be increasing, partly because the eating habits of Asian people have changed similar to Western style. GERD was the main theme of previous IGICS in 2009. Many doctors may have paid attention to GERD since 2009. 

The primary disorder as the origin of upper gastrointestinal bleeding is gastric ulcer (GU) in 4 countries (Japan, Korea, the Philippines, and Thailand). In these countries, the second most occurring is duodenal ulcer (DU). In contrast, DU is the most common in China. There are marked geographical variations in the relative proportion of DU and GU. In Western countries, DU has been more common than GU. However, Groenen et al. showed [11] that the incidence of GU was stable over times, while the incidence of DU declined. Reports from Australia and West Asian countries showed a high DU : GU ratio. The DU : GU ratio in Australia was 6.4 : 1 [12]; in Pakistan, 5 : 1 [13]; India, 17.1 : 1 [14], whereas the Japanese report [15] revealed a low DU : GU ratio. No data are available to explain these results. 

Colon polyps are observed most often by colonoscopy, and hemorrhoid is the first disease as the origin of lower gastrointestinal bleeding in all countries as anticipated. Colorectal cancer is the second most common disorder as both diagnosed and the origin of bleeding. Recently, it is said that the number of patients with colorectal cancer shows a rising trend due to a shift to Western eating habits in East Asian countries. These data support the results of this research. 

CE is selected as the first choice for the diagnosis of small intestinal bleeding. The second choice was DBE. Many respondents left this question blank, suggesting that these new instruments have not come into wide use. Novel developments in CE technology, which is a safe and noninvasive procedure, have made possible the investigation of the small intestine. Many studies [1, 2, 16, 17] have established that small bowel CE is very useful in a variety of clinical situations, including bleeding of obscure origin, detection of small bowel lesions in inflammatory bowel disease (IBD), nonsteroidal anti-inflammatory drug (NSAID) enteropathies, and tumors. 

The clinical application of DBE was reported for the first time in 2001 [18]. DBE has been developed as a new technique where direct observation becomes possible in the entire small intestine. It has been a very useful method for not only quantitative diagnoses [3, 18, 19] but also for endoscopy treatment [20, 21]. Recently, diagnostic performance is becoming excellent for several diagnoses of the small intestine due to the advances of both CE and DBE. The rate for determining the origin of bleeding is from 0% to over 70%. It most likely depends on certain factors such as endoscopic experience and facilities in each hospital.

In this survey, there are no differences in the endoscopic therapy of acute variceal and nonvariceal upper GI bleeding among countries. Most physicians choose EVL as first-line treatment for the patients with esophageal varices rupture. EIS is selected as the second, but those who do not have the technical skill for EIS may select S-B tube. In the case of bleeding gastric ulcer with exposed vessel, clipping is selected as the first choice in 4 countries as was expected. HSE injection is chosen as the second most preferred treatment. Meta-analysis [22–24] confirmed that endoscopic therapy is effective in achieving primary hemostasis in the treatment of bleeding peptic ulcers. It is said that combined therapy with clipping and HSE injection is superior to epinephrine alone for hemostasis in active bleeding and nonbleeding visible vessels [24]. Furthermore, rebleeding was reduced with continuous injection of PPI after endoscopy as compared with placebo or nontreatment. Combinations such as clipping, HSE, electrocoagulation, and infusion of PPI are thought to be used in the clinical setting. 

The number of physicians who choose EMR is larger than those selecting ESD in the treatment of both gastric adenoma and colonic flat adenoma. However, many questions were left blank. EMR and ESD are two techniques [25] designed to remove a large volume of tissue and are targeted toward mucosal early cancer or submucosal lesions of the esophagus [26, 27], stomach [8, 9, 28], and colon [7, 10, 29]. In general, ESD techniques are more difficult, associated with increased complications including perforation or bleeding. In retrospective analysis, ESD is significantly more likely to result in higher en bloc resection rates and higher rates of being recurrence-free at 5 years in early gastric cancer [30]. Another report [31] shows that EMR was as effective as ESD in lesions smaller than 1.5 cm in mucosal gastric neoplasm. Oka et al. reported [32] that ESD was superior to EMR in all size categories, but was associated with a higher odds ratio of perforation and bleeding in early gastric cancer. ESD has been used as a standard endoscopic therapeutic method for upper GI lesions, especially the stomach, whereas colorectal ESD is currently in the developing stage. Scope handling and control are more difficult in the colorectum than the upper GI tract, due to the presence of many folds and flexures and its length. In clinical practice, methods selected from EMR, ESD, and surgical operation may depend on the characteristics of the lesion, technical skill of the endoscopist, and facilities in each hospital.

There are some potential biases in this study. First, the duration of endoscopic experience of the physicians participating in this survey is relatively short in all countries. One-third of them have less than 5 years experience, and it is conjectured that half have been performing endoscopy for less than 10 years. There are many residents and trainers in this survey as previously described. Many physicians answered 0 to the questions 16 and 20, which means they have not used endoscopic treatment to early gastric or colonic cancer patients in a given week. Therefore, the results of this survey may in part not reflect the exact present situation. 

In conclusion, new instruments including CE and DBE have not come into wide use yet, and ESD may be performed only in limited number of hospitals. A standard protocol of endoscopic diagnosis and treatment in patients with GI disease will be needed in the near future. In addition, cooperation to develop skills for more effective management on endoscopy in all Asian countries is necessary.

## Figures and Tables

**Table 1:** Duration of endoscopy experience by participating physicians in each country.

			(Years)				
	<5	5–10	11–15	16–20	>20	N/A	Total
China	20	12	6	3	4	6	51
Indonesia	19	20	11	8	14	3	75
Japan	37	57	40	21	25	10	190
Korea	34	12	8	4	1		59
Philippine	9	4	6	5	5	3	32
Thailand	51	24	7	11	8	6	107

**Table:** (a)

	1	2	3
China	Gastritis	Duodenal ulcer	Gastric ulcer
Indonesia	Gastritis	Gastric ulcer	Esophageal varices
Japan	Gastritis	Reflux esophagitis	Gastric ulcer
Korea	Gastritis	Reflux esophagitis	Gastric ulcer
Philippine	Gastritis	Reflux esophagitis	Gastric ulcer
Thailand	Gastritis	Gastric ulcer	Reflux esophagitis

**Table:** (b)

	1	2	3
China	Duodenal ulcer	Gastric ulcer	Esophageal varices
Indonesia	Esophageal varices	Gastric ulcer	Duodenal ulcer
Japan	Gastric ulcer	Duodenal ulcer	Gastric ulcer
Korea	Gastric ulcer	Duodenal ulcer	Esophageal varices
Philippine	Gastric ulcer	Duodenal ulcer	Esophageal varices
Thailand	Gastric ulcer	Duodenal ulcer	Esophageal varices

**Table:** (c)

	1	2	3
China	Colon polyps	Colorectal cancer	Ulcerative colitis
Indonesia	Colorectal cancer	Ulcerative colitis	Colon polyps
Japan	Colon polyps	Colorectal cancer	Ischemic colitis
Korea	Colon polyps	Colorectal cancer	Ulcerative colitis
Philippine	Colon polyps	Colorectal cancer	Ulcerative colitis
Thailand	Colon polyps	Colorectal cancer	Ulcerative colitis

**Table:** (d)

	1	2	3
China	Hemorrhoids	Colorectal cancer	Colon polyps
Indonesia	Hemorrhoids	Colorectal cancer	Ulcerative colitis
Japan	Hemorrhoids	Diverticula	Colorectal cancer
Korea	Hemorrhoids	Colorectal cancer	Ischemic colitis
Philippine	Hemorrhoids	Colorectal cancer	Diverticula
Thailand	Hemorrhoids	Colorectal cancer	Diverticula

**Table:** (e)

	1	2
China	Double-balloon endoscopy	Angiography
Indonesia	Capsule endoscopy	Angiography
Japan	Capsule endoscopy	Double-balloon endoscopy
Korea	Capsule endoscopy	Angiography
Philippine	Angiography	Scintigraphy
Thailand	Capsule endoscopy	Double-balloon endoscopy

**Table:** (a)

	1	2
China	Sengstaken-Blakemore (S-B) tube	EVL
Indonesia	EVL	EIS
Japan	EVL	EIS
Korea	EVL	Sengstaken-Blakemore (S-B) tube
Philippine	EVL	EIS
Thailand	EVL	EIS

EVL: endoscopic variceal ligation, EIS: endoscopic injection sclerotherapy.

**Table:** (b)

	1	2
China	EMR	Surgical operation
Indonesia	EMR	Hot biopsy
Japan	ESD	EMR
Korea	ESD	EMR
Philippine	EMR	Hot biopsy
Thailand	EMR	Surgical operation

EMR: endoscopic mucosal resection, ESD: endoscopic submucosal dissection.

**Table:** (c)

	1	2	3
China	Clipping	HSE	Injection
Indonesia	HSE	Injection	Clipping
Japan	Clipping	HSE	Electrocoagulation
Korea	Clipping	Electrocoagulation	HSE
Philippine	HSE	Clipping	Injection
Thailand	Clipping	HSE	Electrocoagulation

HSE: local injection of hypertonic saline plus epinephrine therapy.

Injection: intravenous injection of proton pump inhibitor or H_2_ receptor antagonist.

**Table:** (d)

	1	2
China	Surgical operation	ESD
Indonesia	Surgical operation	ESD
Japan	ESD	EMR
Korea	ESD	EMR
Philippine	EMR	Hot biopsy
Thailand	EMR	Surgical operation

EMR: endoscopic mucosal resection, ESD: endoscopic submucosal dissection.

## Appendix

### Contents of Endoscopic Diagnosis and Treatment-Related Questionnaire

1. How many outpatients do you see in a week? (Circle one)
  0–10 10–19 20–29 30–39 40–49 
  50–59 60–69 70–79 80–89 90–99 >100 (number: patients)
2. On how many patients do you perform upper gastrointestinal endoscopy in a week? (Circle one)
  0–10 10–19 20–29 30–39 40–49 
  50–59 60–69 70–79 80–89 90–99 >100 (number: patients)
3. On how many patients do you perform colonoscopy in a week? (Circle one)
  10–19 20–29 30–39 40–49 50–59 
  60–69 70–79 80–89 90–99 >100 (number: patients)
4. On how many patients do you perform endoscopic ultrasonography (EUS) in a month? (Circle one)
  0 1-2 3-4 5–9 10–20 >20 (number: patients)
5. On how many patients do you perform double-balloon endoscopy (small intestine) in a month? (Circle one)
  0 1-2 3-4 5–10 >10 (number: patients)
6. On how many patients do you perform capsule endoscopy in a month? (Circle one)
  0 1-2 3-4 5–10 >10 (number: patients)
7. What kind of disorders do you often diagnose by upper gastrointestinal endoscopy? (Choose three in order)
  (1)  esophageal varices
  (2) esophageal cancer
  (3) reflux esophagitis
  (4) gastritis
  (5) gastric ulcer
  (6) astric cancer
  (7) duodenal ulcer
  (8) others (   )
  No. 1 ()
  No. 2 ()
  No. 3 ()
8. What kind of disorders do you find most often as the origin of upper gastrointestinal bleeding? (Choose three in order)
  (1) esophageal varices
  (2) esophageal cancer
  (3) reflux esophagitis
  (4) Mallory-Weiss syndrome
  (5) gastric ulcer
  (6) gastric cancer
  (7) duodenal ulcer
  (8) Acute gastritis (AGML)
  (9) others (   )
  No. 1 ()
  No. 2 ()
  No. 3 ()
9. What kind of disorders do you often diagnose by colonoscopy? (Choose three in order)
  colorectal cancer
  ulcerative colitis
  Crohn's disease
  ischemic colitis
  intestinal tuberculosis
  colon polyps
  others (   )
  No. 1 ()
  No. 2 ()
  No. 3 ()
10. What kind of lower gastrointestinal disorders do you find most often as the origin of lower gastrointestinal bleeding? (Choose three in order)
  (1) colorectal cancer
  (2) ulcerative colitis
  (3) Crohn's disease
  (4) ischemic colitis
  (5) diverticula
  (6) colonic polyps
  (7) hemorrhoids
  (8) others (   )
  No. 1 ()
  No. 2 ()
  No. 3 ()
11. How do you diagnose bleeding of the small intestine (jejunum and ileum)? (Choose two in order)
  (1) capsule endoscopy
  (2) double-balloon endoscopy
  (3) Scintigraphy
  (4) angiography
  (5) others (   )
  No. 1 ()
  No. 2 ()
12. In what percentage of patients can you not find the origin of gastrointestinal bleeding endoscopically? (Circle one)
  5 10 15 20 25 30 25 40 45 50
  55 60 65 70 >75 (% of total patients with gastrointestinal bleeding)
13. How do you treat patients with rupture of esophageal varices? (Choose two in order)
  (1) endoscopic injection sclerotherapy (EIS)
  (2) endoscopic variceal ligation (EVL)
  (3) Sengstaken Blakemore (S-B) tube
  (4) surgical operation
  (5) others (   )
  No. 1 ()
  No. 2 ()
14. How do you treat patients with gastric adenoma of 2 cm in diameter? (Choose two in order)
  (1) endoscopic mucosal resection (EMR)
  (2) endoscopic submucosal dissection (ESD)
  (3) hot biopsy
  (4) laser therapy
  (5) surgical operation
  (6) others (   )
  No. 1 ()
  No. 2 ()
15. In Japan, early gastric cancer is defined as tumor cells localized within the mucosal or submucosal layer, and not invading the muscular layer or under. Do you agree with this concept? (Circle one)
  Yes
  No
16. How often do you use endoscopic treatment to early gastric cancer patients in a month? (Circle one)
  0 1-2 3-4 5–9 10–14 15–20 >20 (number: patients)
17. How do you treat patients with bleeding from exposed vessels in gastric ulcer? (Choose three in order)
  (1) local injection of absolute ethanol therapy
  (2) local injection of hypertonic saline plus epinephrine therapy
  (3) clipping
  (4) electrocoagulation
  (5) heater probe coagulation
  (6) local application of hemostatics including fibrin glue
  (7) intravenous injection of proton pump inhibitor or H2 receptor antagonist
  (8) surgical operation (including laparoscopic operation)
  (9) others (   )
  No. 1 ()
  No. 2 ()
  No. 3 ()
18. How do you treat patients with flat adenoma of 3 cm in diameter? (Choose two in order)
  (1) endoscopic mucosal resection (EMR)
  (2) endoscopic submucosal dissection (ESD)
  (3) hot biopsy
  (4) laser therapy
  (5) surgical operation (including laparoscopic operation)
  (6) others (   )
  No. 1 ()
  No. 2 ()
19. In Japan, early colorectal cancer is defined as tumor cells localized within the mucosal or submucosal layer, and not invading the muscular layer or under. Do you agree with this concept? (Circle one)
  Yes
  No
20. How often do you use endoscopic treatment to early colorectal cancer patients in a month? (Circle one)
  0 1-2 3-4 5–9 10–14 15–20 >20 (number: patients)
